# Cannulation Navigation: Accreditation-Informed Process Redesign to Improve ECMO Transportation Efficiency

**DOI:** 10.1016/j.jaccas.2026.110212

**Published:** 2026-09-16

**Authors:** Wynne Lok, Sandra G. Page, Heather J. Price, Katie A. Rodwell

**Affiliations:** Department of Heart and Vascular Service, UNC Health Rex, UNC Health, Raleigh, North Carolina, USA

**Keywords:** collaboration, extracorporeal membrane oxygenation, interfacility transfer, quality improvement, transport, workflow

## Abstract

**Background:**

Patients requiring emergency extracorporeal membrane oxygenation (ECMO) support at UNC Health Rex—54% of whom presented with myocardial infarction—faced prolonged and inconsistent transfer times to the UNC Medical Center.

**Project Rationale:**

A lack of standardized workflow contributed to variable response times, ambiguity around transportation responsibilities, and inefficiencies that could not meet the urgency required for ECMO candidates.

**Project Summary:**

This project used registry data to identify gaps, convened a multidisciplinary team to set goals, streamlined communication, revised protocols, optimized workflows, and targeted education to improve early recognition, coordination, and transfer efficiency for ECMO patients.

**Take-home Message:**

Despite challenges with aligning multiple specialties, changing long-standing habits, and coordinating resources across campuses, the team significantly reduced transfer times and created a sustainable model that strengthened systemwide care for critically ill ECMO patients.

## Visual Summary

**Figure:**
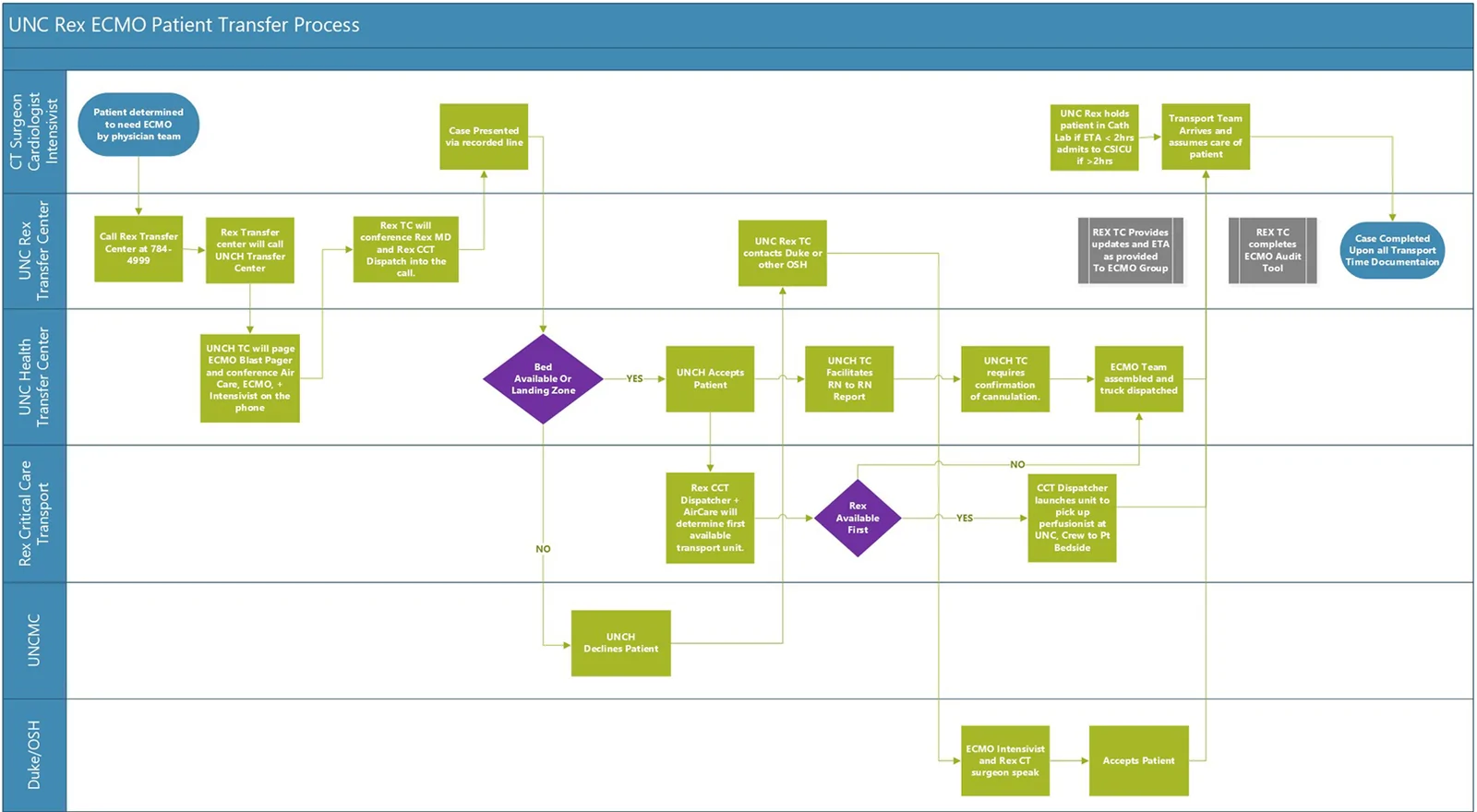

## Background

Extracorporeal membrane oxygenation (ECMO) is a pump to support the cardiac and respiratory systems during a critical situation, but there can be complications in up to 30% of ECMO patients during interfacility transportation.^1^ In facilities with low ECMO transfers, optimizing protocols and teamwork can improve chances for favorable outcomes.^2^ Standardizing the process does not have to be costly to improve survival and complication rates.^3^ Patients requiring emergency ECMO support (54% of which were diagnosed with ST-segment elevation myocardial infarction or non–ST-segment elevation myocardial infarction) at the UNC Health Rex (Rex) faced prolonged and inconsistent transfer times to the UNC Medical Center (UNCMC), largely because of fragmented communication, delayed acceptance decisions, transportation availability issues, and delays in mobilizing ECMO specialists and equipment.

## Project Rationale

Delays created unnecessary risk for patients with rapidly deteriorating cardiopulmonary status, including extended ischemic time and added stress for frontline teams attempting to coordinate care across multiple departments and campuses. The lack of a standardized, reliable workflow resulted in variability in response times, uncertainty about transportation responsibility, and inefficiencies that could not support the urgency required for ECMO candidates. The team sought to address these gaps by redesigning the ECMO transfer process to ensure faster, safer, and more predictable transfers for critically ill patients.

## Project Description

The project used the Chest Pain–MI Registry, CathPCI Registry, and transfer records to create a list of potential patients based off the cardiogenic shock and mechanical device data points, to observe trends in patient history or procedures and to provide transfer times. Being able to retrieve key measures such as door-to-device times, first medical contact to intervention, and outcomes from the Chest Pain–MI Registry and CathPCI Registry provided further evidence that work needed to be completed.

A multidisciplinary work group was assembled, leveraging experience from the chest pain center accreditation process. Members included directors and managers across clinical areas, interventional cardiologists, cardiac surgeons, intensivists, respiratory therapists, perfusionists, transfer center personnel, Rex critical care transport (CCT), pharmacy, blood bank, and advanced practice providers to allow everyone a chance to bring ideas and contribute their voice.

After discussions with the transfer centers, the UNCMC managers and providers, and the heart and vascular director at Rex, a single multidisciplinary call was established to connect Rex and the UNCMC teams upon patient identification. This ensured rapid consensus that the patient was an ECMO candidate and allowed immediate acceptance.

The ECMO order set was updated specifically for Rex to include additional blood for transportation and an intensivist consult in case the patient was transferred to the intensive care unit (ICU) before transportation. Protocol changes clarified various job roles for the CCT crews, dispatch team, and providers participating in the care of ECMO patients, thereby reducing confusion and alignment gaps during emergency transfers.

Previously, Carolina Air Care, the UNCMC's transportation team, was the only team transporting ECMO patients. Carolina Air Care and CCT established an agreement in which the closest available ambulance would perform the transportation. Handheld blood analysis machines were added to each ambulance, which aided staff in monitoring patients and adjusting care. Funding from the UNC Health Foundation was requested to cover the machine costs outside the midyear budget without delaying until the next capital cycle.

A contingency workflow was created that allowed ECMO transfers to proceed even if an ICU bed was temporarily unavailable at the UNCMC. In these rare cases, the plan placed the patient in the cardiac catheterization laboratory with the ECMO specialist and ICU nurse until a bed was ready.

Because ECMO specialists were located at the medical center, the updated workflow allowed the ECMO specialist to disengage quickly, collect the ECMO bag and pump, and meet transportation at the pickup location within 15 minutes. CCT dispatched a van to pick up the ECMO specialist and transport them directly to Rex.

Changes were made to the electronic health record software to optimize documentation for staff. During cardiac catheterization procedures, a pause was added to ensure that patients were identified early and appropriate interventions were performed. An ECMO-specific index was built so the teams could access health data immediately from the patient's chart or in secure chats to help with their decisions and transportation.

Education enhanced early recognition and confidence in caring for ECMO patients. Nursing educators developed an LMS (learning management system) module to send out to all relevant staff. The CCT and cardiac surgery ICU completed annual ECMO skills. The UNCMC provided an ECMO specialist to host a virtual presentation for CCT on ECMO transportation. Mock drills were completed for cannulating and transporting patients, starting from the Rex cardiac catheterization laboratory and ending at the UNCMC.

## Project Deliverables

Data were obtained from Chest Pain–MI Registry, CathPCI Registry, and transfer logs to track times and the ways these times were affected. Before implementing workflow changes, 10 patients underwent transfer in 1 year; within 7 months of the new process, 6 patients had been transferred. CCT conducted 100% of the transportation, eliminating delays tied to external resource availability that had previously occurred. The transportation time for arrival at the facility after deciding on ECMO was reduced from 3 hours 47 minutes to 1 hour 23 minutes (Table 1). Overall transfer times fell from 5 hours 47 minutes to 4 hours 35 minutes. An independent, unequal, 1-tailed *t*-test with a significance level of 0.05 demonstrated a statistically significant improvement (*P* = 0.0108), supporting a 98.92% confidence level that the interventions reduced transfer times.

**Table 1:** Average Transportation Times

	Prior State, h:min	Current State, h:min
MD call to dispatch	2:06	0:56
Initial transportation to dispatch	1:40	0:21
MD call to arrival at Rex	3:47	1:23
Initial transportation to arrival at Rex	3:20	0:49
Arrival at Rex to patient ready	1:09	2:38
Overall time	5:47	4:35

MD = physician; Rex = UNC Health Rex.

## Project Outcomes

Through the changes made, the hospital had been able to identify potential ECMO patients early and either send them more quickly before they needed cannulation or cannulating them and sending them faster to a higher level of care that could address their issues, which improved patient outcomes (Table 2). The rise in the percentage of venoarterial ECMO potentially demonstrated that the organization recognized the need for ECMO sooner in respiratory patients and transferred the patients out before needing venovenous ECMO.

**Table 2:** Project Outcomes

Outcome	Prior State	Current State
Door-to-cannulation time, h:min	3:55	3:40
Percentage of VA ECMO	50%	66.67%
Percentage mortality during stay	30%	33.30%
Percentage of VA ECMO mortality during stay	60%	25%

VA ECMO = venoarterial extracorporeal membrane oxygenation.

With the clarification of roles and responsibilities, staff and providers found the process more efficient, as they knew who and how to contact for an ECMO patient, including who was placing what lines and who oversaw the patient and orders. Staff felt less stressed after education and fixing lack of access to key transfer tools to care for ECMO patients.

Other changes included reducing redundant steps, improving transportation coordination, and minimizing unnecessary use of multiple teams to lower overall system costs and payers. The drop in the percentage of venoarterial ECMO patients who expired after transfer potentially demonstrated how earlier transfers for treatment reduce mortality. Workflow changes from the project decreased the risk profile for high-acuity transfers through education and early transfers.

## Discussion

Chest pain center accreditation status enhanced the facility's ability to recognize the issue, analyze the workflow, build on the collaboration created from accreditation, and improve resuscitation efforts for the organization. By shortening the time to mechanical circulatory support and intervention, the project improved metrics such as door-to-cannulation time, early recognition and transfer of respiratory patients, and cardiogenic shock outcomes. These improvements supported accreditation goals for rapid identification of high-risk patients, reduced treatment delays, strengthened transfer protocols, and enhanced care continuity. The new workflow was embedded in routine operations and represented a major advancement in system-wide ECMO care.

The project faced multiple challenges, both expected and unexpected. With the number of hands involved with ECMO patients, it was intricate work aligning the various specialties and molding previous practices to best practices. Working with 2 separate hospitals was demanding given the available resources and policy differences. There was also variability in naming, which came as a surprise when the project team would talk about “the perfusionist” and the UNCMC used the term “ECMO specialists.” The project also faced liability barriers when attempting to expedite the ECMO specialists' movement from the UNCMC to Rex.

Additional work continues, focusing on early recognition as well as improving transportation times by providing better transportation methods to bring the ECMO specialist to Rex. Although the data show improvement, data need to continue to be tracked over a longer period of time to monitor sustainability. More work needs to be done to calculate the economic impact of these changes. Because of early recognition, fewer cannulations were completed at Rex, thus reducing procedure and transportation costs. The time saved from the project reduced delays in cases planned for the catheterization laboratory and operating rooms as well as time taken from staff assisting with the cannulation and transportation.

## Conclusions

Through targeted workflow redesign, multidisciplinary collaboration, and education, Rex reduced ECMO interfacility transfer times from 5 hours 34 minutes to 4 hours 35 minutes. Continued efforts aim to improve equipment and staffing logistics, and the work has stimulated additional quality improvement initiatives around cardiogenic shock and pulmonary embolism care. Not every hospital is able to care for ECMO patients or ill patients beyond their resources, and this work could provide a reference for hospitals who need to transfer patients in a timely manner.

**Visual Summary:**
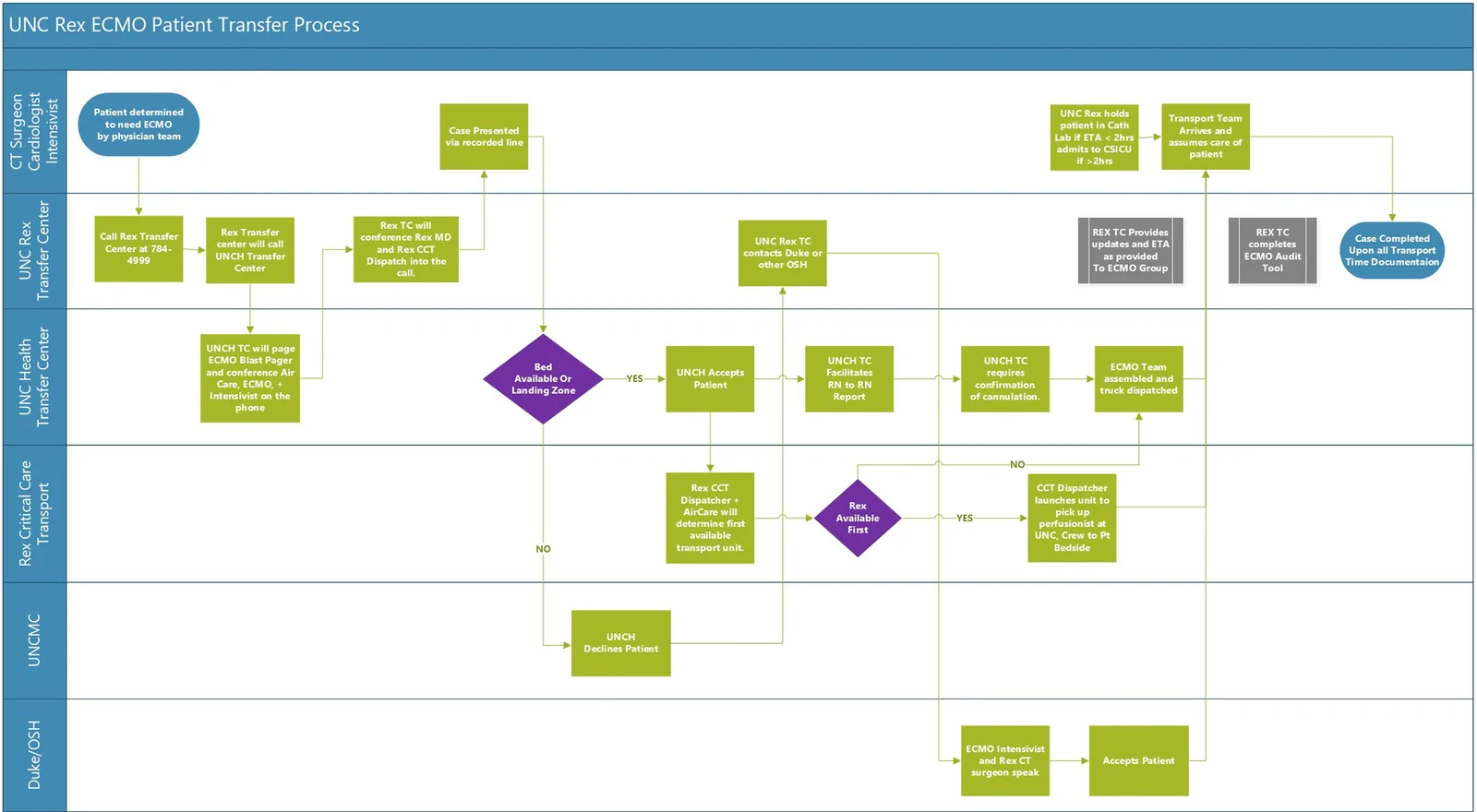
Swim Lane Chart of Workflow After Changes The swim lane chart captures the workflow after all the changes from the project. Blue ovals denote start/end points; green boxes denote tasks or activities; purple diamonds denote decision points; and gray squares denote side tasks completed at the same time. Cath lab = cardiac catheterization laboratory; CCT = critical care transport; CSICU = cardiac surgery intensive care unit; CT = cardiothoracic; ECMO = extracorporeal membrane oxygenation; eta = estimated arrival time; MD = doctor; OSH = outside hospital; pt = patient; Rex = University of North Carolina Health Rex; RN = nurse; TC = transfer center; UNCH = University of North Carolina–Chapel Hill (UNCMC); UNCMC = UNC Medical Center.

## Funding Support and Author Disclosures

Funding to equip ambulances with handheld wireless devices for blood analysis was provided by the UNC Health Foundation. The authors have reported that they have no relationships relevant to the contents of this paper to disclose.

**Take-Home Messages**

• Patients requiring extracorporeal membrane oxygenation (ECMO) support—many presenting with myocardial infarction—faced unsafe delays and inconsistent workflows during interfacility transfers, prompting the development of a multidisciplinary process to accelerate decision-making, transportation coordination, and early clinical recognition.
• Despite challenges with aligning multiple specialties, changing long-standing habits, and coordinating resources across campuses, the team significantly reduced transfer times and created a sustainable model that strengthens systemwide care for critically ill ECMO patients.
